# Venous Doppler ultrasound in critically Ill COVID-19 patients: game changer in anticoagulation therapy

**DOI:** 10.1186/s13089-020-00201-7

**Published:** 2020-12-28

**Authors:** Marta Alfageme, Jorge González Plaza, Santiago Méndez, Juan A. Gómez Patiño, María L. Collado, José M. Abadal, Rocío González Costero, Teresa Fontanilla, Agustín García Suárez

**Affiliations:** 1 Department of Interventional Radiology, Hospital Universitario Puerta de Hierro, Majadahonda, España; 2 Department of Interventional Radiology, Hospital Universitario Severo Ochoa, Leganés, España; 3 Department of Radiology, Hospital Universitario Puerta de Hierro, Majadahonda, España

## Abstract

**Background:**

COVID-19 infection has been associated with a high rate of thrombotic events, such as deep vein thrombosis (DVT) and acute pulmonary embolism (APE).

**Methods:**

The purpose of our retrospective study was to evaluate the prevalence of asymptomatic DVT in lower limbs in critically ill COVID-19 patients (*n* = 23) with severe respiratory failure and high levels of D-dimer by bedside Doppler ultrasound (DU).

**Results:**

DVT was diagnosed in 14 cases (60.87%), 5 in proximal venous territory and 9 in infrapopliteal veins. Computed Tomography Pulmonary Angiography (CTPA) was performed in six patients and all of them showed acute pulmonary embolism (APE) at segmental or subsegmental branches of pulmonary arteries. These patients (APE or DVT confirmed) were treated with therapeutic doses of anticoagulant therapy.

**Conclusion:**

In critically COVID-19 ill ICU patients with severe respiratory failure and elevated D-dimer, the incidence of asymptomatic DVT is high. We propose that DU allows detection of DVT in asymptomatic patients, adding a factor that may balance the decision to fully anticoagulate these patients.

## Introduction

COVID-19 infection has been associated with a high rate of thrombotic events, such as deep vein thrombosis (DVT) and acute pulmonary embolism (APE) in patients admitted to intensive care unit (ICU) [1].

Several studies report a high risk of DVT in COVID-19 critically ill patients with pneumonia despite adequate thromboprophylaxis [2] and have suggested the use of anticoagulant therapy in therapeutic range [3].

The purpose of our study was to evaluate the prevalence of asymptomatic DVT in lower limbs in critically ill COVID-19 patients with severe respiratory failure and high levels of D-dimer by bedside Doppler ultrasound (DU).

## Materials and methods

This is an observational descriptive retrospective study performed at a third level hospital in Madrid between the 1st and 10th of April 2020. DU was performed on all consecutive confirmed COVID-19 patients admitted to the Intensive Care Unit (ICU) with the diagnosis of severe pneumonia (with radiological pattern compatible with COVID-19 and extensive involvement in chest X-ray), who developed acute severe respiratory failure, requiring invasive mechanical ventilation and with elevated D-dimer levels > 0.5 µg/ml (normal range < 0.5 µg/ml).

Computed tomography pulmonary angiography (CTPA) could not be performed due to the risk of unstable patient transfer to the radiology, so DU was carried out at patient bedside to screen venous thromboembolic disease.

Nevertheless, later respiratory and clinical improvement of 6 patients permitted CTPA.

Patients were excluded if they were under full-dose anticoagulant therapy or if they developed symptomatic DVT.

Several patients showed alterations in coagulation parameters, so International Society of Thrombosis and Haemostasis (ISTH) score, a scoring system for disseminated intravascular coagulation (DIC), was calculated.

All patients at ICU received thromboprophylaxis with subcutaneous low molecular weight heparin (LMWH) (enoxaparin 40 mg/24 h), adjusted for renal insufficiency (creatinine clearance < 30 ml/min) and overweight patients (> 100 Kg).

**Fig. 1:**
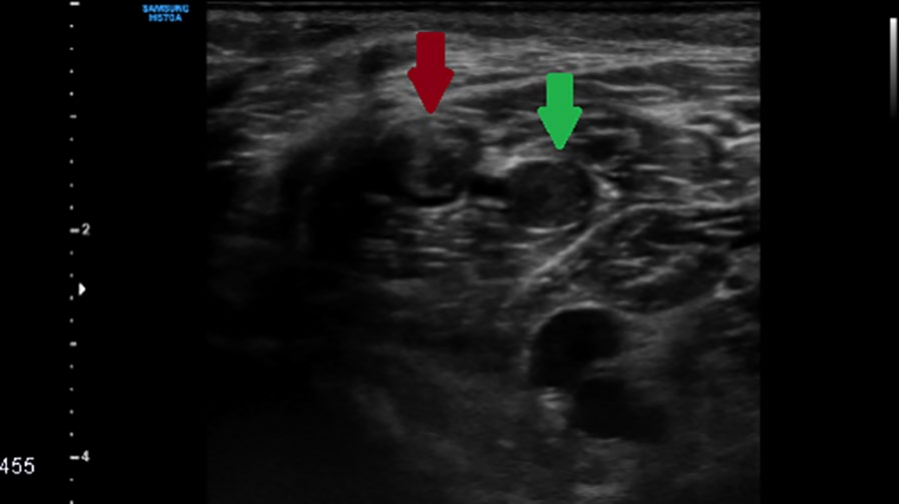
Gastrocnemius veins thrombosis. Echogenic content is identified within the veins. The content is better visualized in the vein on the left (red arrow) than in the right (green arrow)

**Fig. 2:**
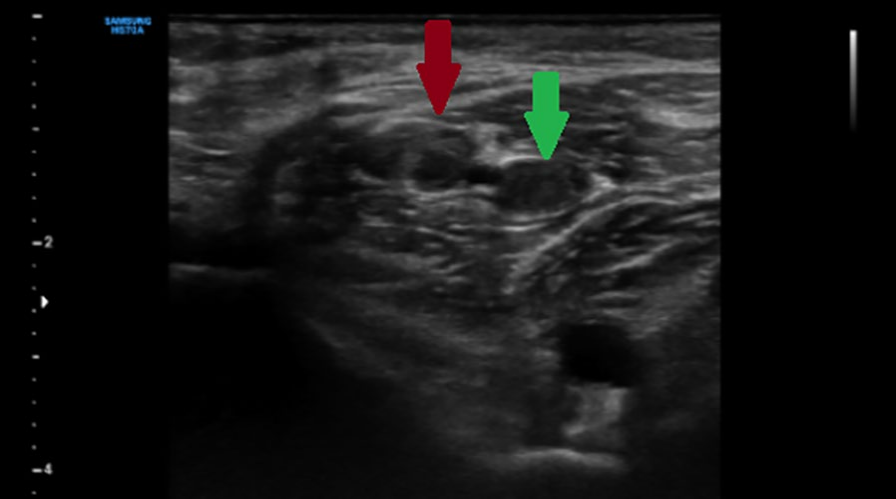
Absence of collapse after compression with the ultrasound transducer. With the transducer in the same position as Fig. 1, pressure is exerted. In the absence of thrombosis, the veins should collapse. In this case, they not collapse (arrows). This maneuver helps to confirm thrombosis of the right side vein (green arrow)

**Fig. 3:**
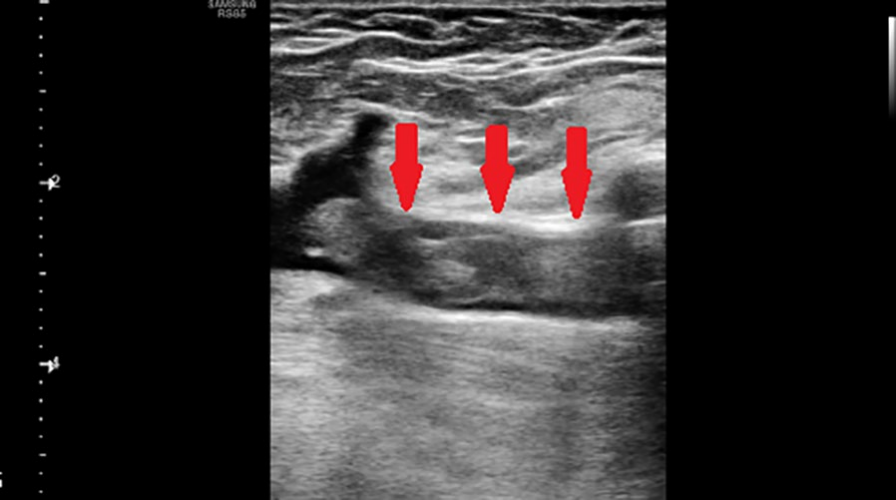
Longitudinal image of the posterior tibial vein. It is enlarged and with echogenic content in relation to acute venous thrombosis (arrows)

DU examinations were practiced by two radiologists with more than 10-year of experience (Samsung HS50 ® equipment US and with a multifrequency linear probe). DU protocol includes grayscale, color and spectral Doppler evaluation. The examination included the deep venous system of the thigh, calf and both saphenous. DVT was diagnosed in cases of increased vein diameter, presence of echogenic material, non-compressibility of the veins and absence of color and spectral Doppler signal (Figs. 1, 2, 3).

Formal approval from the Medical Ethics Review Committee was not required as the Medical Research Involving Human Subjects Act (WMO) did not apply for this observational study.

Regarding descriptive statistical analysis, categorical data were expressed as relative and absolute frequencies, while numerical data were reported as the mean and standard deviation (SD) if there was a normal distribution or median and interquartile range (IQR) if they have a non-normal distribution. Qualitative variables were presented by the frequency distribution and percentages.

## Results

Initially, we had included 27 patients, but we excluded 4 patients from this study: 2 of them because they were already treated with therapeutic anticoagulant therapy and other 2 patients as they developed symptomatic DVT. Thus, the study comprised 23 patients, 16 males (69.57%) and 7 females (30.43%). Mean patient age was 59.17 ± 12.9 (DS).

DVT was diagnosed in 14 cases (60.87%) (Table 1). Five in proximal venous territory and nine in infrapopliteal veins. No proximal and distal thrombosis was found simultaneously in the same patient.

**Table 1:** Characteristics of COVID-19 patients included in the study

AGE (years)	SEX^a^	DVT^b^	D-DIMER (µg/ml)	ISTH score	CTPA^c^
55	1	1	13.99	2	1
34	1	1	18.47	3	n/a
51	1	0	10.11	2	n/a
64	1	0	2.94	1	n/a
57	1	1	93.24	3	1
48	1	1	12.14	3	n/a
77	1	1	49.88	3	1
59	0	1	59.60	3	1
73	1	1	12.08	1	n/a
43	0	1	8.74	1	1
63	0	1	15.07	2	n/a
53	1	0	3.25	1	n/a
41	1	0	13.82	3	n/a
72	0	1	3.51	1	n/a
76	1	1	57.48	2	n/a
75	1	0	9.30	3	n/a
64	1	1	12.31	2	1
49	0	0	4.60	4	n/a
72	1	1	18.79	3	n/a
52	0	0	4.79	2	n/a
71	0	0	4.30	1	n/a
71	1	1	17.26	3	n/a
41	1	0	13.82	1	n/a

All patients presented extensive and bilateral lung involvement on Chest-X-Ray

*DVT* deep venous thrombosis, *ISTH* International Society of Thrombosis and Hemostasis Criteria for Disseminated intravascular Coagulation (DIC), *ICU* Intensive Care Unit

^a^Sex: 1 = male, 0 = female, ^b^DVT: 1 = positive, 0 = negative ^c^Computed Tomography Pulmonary Angiography: 1 = positive, 0 = negative, n/a = not available

CTPA was performed in six patients and all of them showed acute pulmonary embolism (APE) at segmental or subsegmental branches of pulmonary arteries. None of them showed thrombus in the main or lobar pulmonary arteries. DU confirmed DVT in these 6 cases. In five of them the thrombosis was distal.

The median of D-Dimer was 12.31 µgr/ml (IQR 6.75–17.86 µg/ml). Patients with DVT had higher D-Dimer 16.16 µg/ml (IQR 12.18–42.03) µg/ml than patients without DVT 4.79 µg/ml (IQR 4.3–10.11) µg/ml.

All the patients presented ISTH score < 5, suggesting non-overt or low grade DIC (Table 1).

Patients with APE (n = 6) or confirmed DVT were treated with therapeutic doses of anticoagulant therapy (1 mg of enoxaparin/kg every 12 h), adjusted to weight and renal function.

Major hemorrhagic complications occurred in 3/23 patients (13%), two of them developed inferior epigastric artery bleeding and one patient presented middle hemorrhoidal artery bleeding, requiring in all cases endovascular embolization. One of the patients with epigastric bleeding had infrapopliteal thrombosis. The other two patients had proximal DVT.

## Discussion

COVID-19 has been associated with a higher risk of thrombotic events like DVT and APE [4–6]. Some studies propose that thromboembolic events could be secondary to a systemic procoagulant response (excessive inflammation, hypoxia, platelet activation and endothelial dysfunction) to COVID-19 infection [7, 8]. The evidence to date supports the concept that the thrombotic manifestations of severe COVID-19 are due to the ability of SARS-CoV-2 to invade endotelial cells via ACE-2 (angiotensin-converting enzyme 2), which is expressed on the endothelial cell surface. However, in patients with COVID-19, the subsequent endothelial inflammation, complement activation, thrombin generation, platelet, and leukocyte recruitment, and the initiation of innate and adaptive immune responses culminate in immunothrombosis, ultimately causing (micro)thrombotic complications, such as deep vein thrombosis, pulmonary embolism, and stroke [9]. The frequency of these complications increases in critically ill patients admitted to the ICU [1, 2]. The results of autopsy studies indicate the presence of pulmonary.

endothelial damage and microthrombosis. In a case series of 4 autopsies of COVID-19-infected patients from New Orleans with sudden respiratory decompensation, it was shown that there were no thromboembolisms in the major pulmonary arteries, but small thrombi were present in sections of the peripheral lung parenchyma [10]. It is not clear from the clinical studies whether the thrombotic pulmonary complications are APE or primary pulmonary thrombosis. The presence of distal and microthrombosis in many patients suggests local pathology, which does not exclude the possibility of embolic events [11]. In view of this, some groups recommend full dose anticoagulant therapy for patients with unfavorable evolution and worst prognosis, especially in those with sepsis, elevated levels of D-dimer and disseminated intravascular coagulopathy [3, 12]. Another study found lower mortality rate in critically ill patients under anticoagulant therapy [13].

In our study, we found an increased incidence of asymptomatic DVT (60.87%), mostly in the infrapopliteal venous system in selected COVID-19 patients (elevated D-Dimer and severe respiratory failure). The presence of APE was also documented in the six patients who CTPA was performed. One of the limitations of our study is the lack of CTPA of all the patients.

A recently study also found an increased frequency of distal DVT up to 85% of the COVID-19 ICU patients [5]. Multiple studies reported a high incidence of DVT in critically ill COVID-19 patients, one of them found an incidence of 47%, but all critically patients were included, not only the severe patients [1]. In another study, the incidence was 20% but the authors did not specify whether asymptomatic patients were also studied [2].

Despite our study has limitations as small sample size, the results suggest a higher incidence of asymptomatic DVT in critically ill COVID-19 patients than reported by others studies in non-COVID-19 critically ill patients where the incidence was around 10% [14, 15].

There is not a consensus on the use of anticoagulant therapy on asymptomatic infrapopliteal DVT no COVID-19 patients because of the lower probability of thrombus migration into the lungs and some groups prefer an expectant management [16] while others propose to use anticoagulant therapy (17). The indication of treatment depends on the clinical context of each patient. In our critically ill COVID-19 patients, documenting the DVT helped tip the balance to full anticoagulation.

Therefore, we propose the use of bedside ultrasound to detect DVT including infrapopliteal, especially in ICU patients who cannot be mobilized to perform CTPA.

## Conclusion

In critically COVID-19 ill ICU patients with severe respiratory failure and elevated D-dimer, the incidence of asymptomatic DVT is high.

DU allows detection of DVT in asymptomatic patients, adding a factor that may influence the decision to fully anticoagulate these patients.

## Data Availability

All data used during this study are available by email at the request of the editorial committee.

## Abbreviations

DVT: Deep vein thrombosis
ICU: Intensive care unit
APE: Acute pulmonary embolism
DU: Doppler ultrasound
CTPA: Computed tomography pulmonary angiography
ISTH: International Society of Thrombosis and Haemostasis
DIC: Disseminated intravascular coagulation
LMWH: Low molecular weight heparin
SD: Standard deviation
IQR: Interquartile range
N/A: Not available
